# Acute Liver Failure in Sickle Cell Disease: A Perfect Storm

**DOI:** 10.7759/cureus.15680

**Published:** 2021-06-16

**Authors:** Nicholas B Burley, Kenneth D Miller

**Affiliations:** 1 Internal Medicine, Sinai Hospital of Baltimore, Baltimore, USA; 2 Medical Oncology, Sinai Hospital of Baltimore, Baltimore, USA

**Keywords:** adult sickle cell anemia, sickle cell crisis, liver infarction, sickle cell hbsc, sickle cell complications, sickle cell disease: scd, sickle cell disease complications, congestive hepatopathy, liver and gall bladder disease, rare liver disease

## Abstract

Sickle cell hepatopathy is a well-described but uncommonly seen complication of sickle cell disease and is usually caused by multiple overlapping processes. A more acute liver complication is hepatic sequestration which is important to recognize in order to initiate life-saving treatment. A 33-year-old woman with sickle cell disease complicated by painful crises, splenic infarction and significant alcohol abuse presented with gastrointestinal distress, pain crisis, acute-on-chronic anemia, and hyperbilirubinemia in the setting of greater than baseline alcohol consumption. She was found to have hepatomegaly, encephalopathy, severe jaundice, and severe hyperbilirubinemia. She was treated with red cell exchange and supportive care which resulted in an improvement in her symptoms as well as hyperbilirubinemia. She was discharged with plans for monthly red cell exchange, iron chelation therapy, and close monitoring of liver disease was planned upon discharge. This case illustrates that chronic liver disease can occur in sickle cell disease (Hgb SS) especially in the setting of acquired iron overload. More acutely, sequestration is a serious and life-threatening complication of sickle cell disease that can culminate in acute liver failure. Primary treatment for hepatic sequestration is red cell exchange along with management of contributing comorbidities, and symptomatic management of encephalopathy. In end-stage liver disease, transplantation may be considered in the context of the patient’s clinical status.

## Introduction

Sickle cell disease (SCD) is a common inherited disorder affecting erythrocytes by the formation of hemoglobin S (Hgb S). In physiologically strenuous environments (i.e., deoxygenation, acidemia, extreme cold), Hgb S polymerizes leading to erythrocyte sickling. This deformation precipitates vaso-occlusive crises, hemolysis, reduced nitric oxide bioavailability, inflammation, endothelial injury, and leukocyte and platelet activation [1]. Vaso-occlusive crises may occur in any number of tissues, commonly lungs, brain, bone, kidneys, and spleen [2].

Less commonly, sickle cell crises in the liver can lead to hepatopathy, which is a well-described but uncommonly seen complication of sickle cell disease and is usually caused by multiple concurrent factors [3, 4]. Comorbid conditions often contribute to the development of hepatopathy in sickle cell disease, such as chronic blood transfusions, alcohol abuse, acute and chronic hepatitis B and C infection.

Hepatopathy in patients with sickle cell disease can have acute and severe manifestations, for example hepatic sequestration, vascular occlusion, acute ischemia, and cholestasis can all lead to acute liver failure and it is important to recognize this condition and initiate appropriate management [3-5]. In this case, we discuss a young woman with homozygous sickle cell disease presenting with severe gastrointestinal symptoms due to acute hepatic sequestration.

## Case presentation

A 33-year-old woman with homozygous sickle cell disease complicated by multiple admissions for painful crises and autosplenectomy presented with a two-week history of nausea and vomiting. She also has a history of significant alcohol abuse and recently increased her alcohol intake to approximately 6-8 ounces of vodka daily. She began to have nausea and vomiting along with her more typical sickle cell crisis pain and was found to have profound anemia and hyperbilirubinemia.

**Figure 1:**
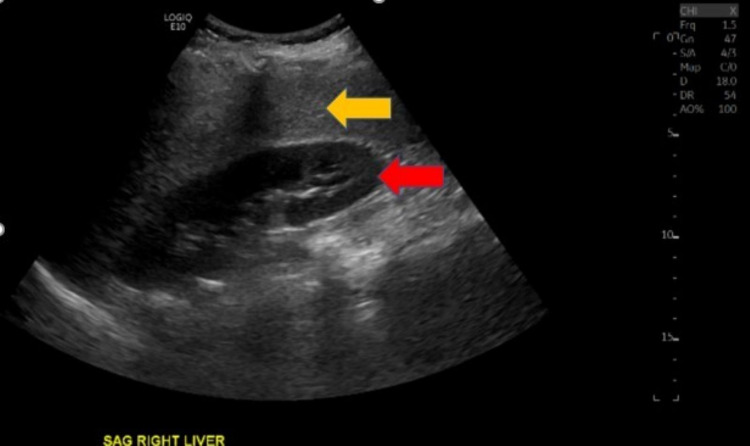
Ultrasound imaging of left liver lobe (sagittal plane) Ultrasound imaging (sagittal plane) of right liver lobe parenchyma (yellow arrow) adjacent to right kidney (red arrow) demonstrating hepatic steatosis.

**Figure 2:**
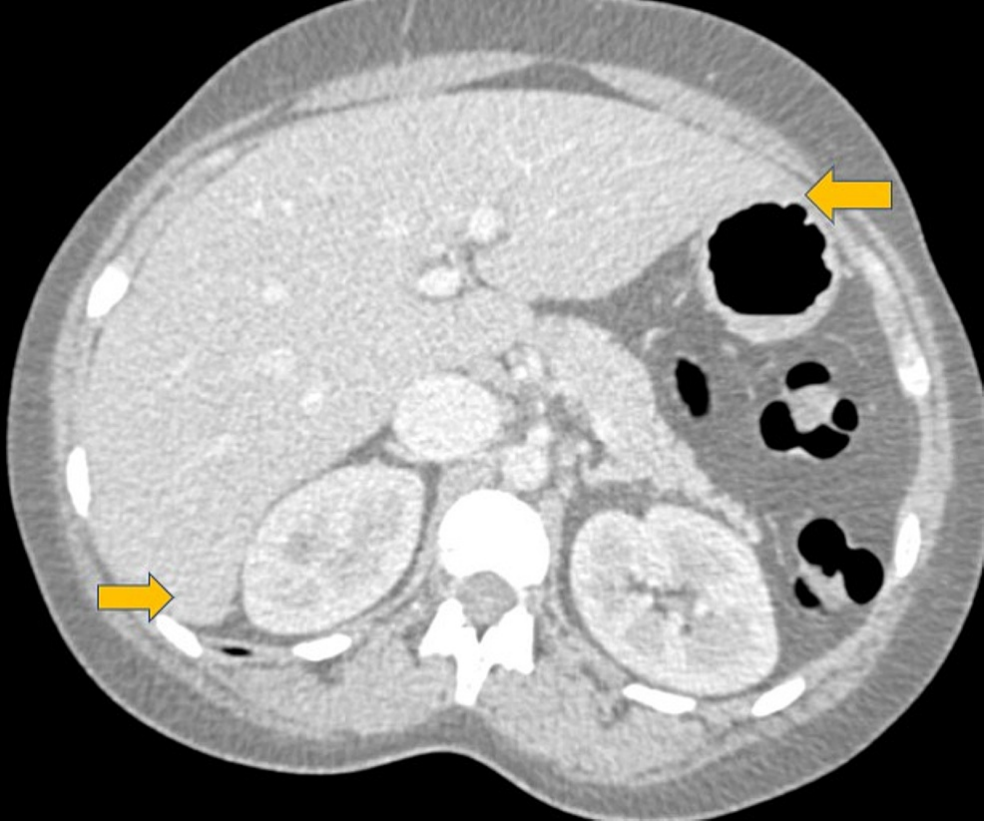
Computed tomography of abdomen Computed tomography of abdomen and pelvis with intravenous contrast demonstrating hepatomegaly with a span of 25 cm (yellow arrows). When compared to prior imaging studies obtained 10 months prior, the patient’s hepatomegaly was a new and significant change.

​Physical examination revealed severe jaundice, hepatomegaly and mild encephalopathy. Pertinent laboratory findings included white blood cell count 33.6 K/mm^3^, hemoglobin 6.2 g/dL, platelets 275 K/mm^3^, pre-exchange hemoglobin S 72.1%, total bilirubin 52.9 mg/dL, direct bilirubin 33.3 mg/dL, alkaline phosphatase 231 units/L, aspartate transaminase (AST) 113 units/L, alanine transaminase (ALT) 32 units/L, haptoglobin <10 mg/dL. Further tests revealed nonreactive HIV antigen and antibody, negative hepatitis serology, negative autoimmune panel, iron level 188 mcg/dL, total iron-binding capacity (TIBC) 233 μg/dL, ferritin 9,636.6 ng/mL, and zinc 41 μg/dL (normal low 56 μg/dL). Ultrasound of the right-upper quadrant (RUQ) revealed hepatic steatosis (Figure 1) and a patent portal vein. CT abdomen (Figure 2) and abdominal MRI findings were consistent with hepatomegaly and iron overload with liver involvement but not cardiac. Her pain did not diminish during the first week of her admission, and she was complaining of increasing RUQ pain and tenderness on her physical examination.

​Treatment included red cell exchange leading to a post-exchange hemoglobin S of 15%, folic acid, hydroxyurea and opioids to manage the acute sickle cell crisis. A liver biopsy was deferred given the patient’s severe coagulopathy (INR 2.6). She was safely discharged from hospital in stable condition and with a reduction of her total bilirubin to 33 mg/dL. Prior to discharge, the patient was referred for alcohol counselling and education. Monthly red cell exchange, iron chelation therapy, and close monitoring of liver disease were planned upon discharge. A year later, in follow-up the patient was found to have tolerated red cell exchanges and iron chelation without any complications or emergent hospitalizations.

## Discussion

The broad designation of sickle cell hepatopathy describes a spectrum of liver disease. Differentiation of sickle cell hepatopathy into more specific conditions may be difficult, however several articles have attempted to delineate acute sickle cell hepatic crisis, acute hepatic sequestration, and acute intrahepatic cholestasis [3, 6].

Acute sickle cell hepatic crisis caused by sinusoidal obstruction, Kupffer cell hypertrophy, and centrilobular necrosis presents with acute right upper quadrant pain, gastrointestinal distress, fever, tender hepatomegaly and jaundice. Laboratory findings include AST, ALT levels that rarely exceed three times the upper limits, total bilirubin levels less than 15 mg/dL, and normal to marginally elevated alkaline phosphatase which can be managed with treatment of SCD crisis [3, 6-9].

Acute hepatic sequestration presents with acute right upper quadrant pain, hepatomegaly, and anemia. Laboratory findings include normal AST, ALT levels, total bilirubin levels up to 24 mg/dL, and severely elevated alkaline phosphatase up to 650 units/L, which can be treated with exchange transfusions. In rare instances, treatment and resolution of acute hepatic sequestration may lead to the release of trapped functional red cells from the hepatic sinusoids leading to severe hypervolemia, thrombosis, and high rates of mortality [3, 6, 9].

Acute intrahepatic cholestasis is the most severe and fatal manifestation of this constellation of diseases with widespread sickling in hepatic sinusoids, ischemia and intracanalicular cholestasis. It presents similar to acute hepatic sequestration compounded by severe jaundice, renal impairment, bleeding diathesis, and encephalopathy. Laboratory findings include severely elevated AST, ALT greater than 1000 units/L, total bilirubin well above 100 mg/dL with conjugated predominance and alkaline phosphatase ranging widely from normal ranges to extreme elevations above 1000 units/L. Other pertinent laboratory findings include renal insufficiency, hyperammonemia, hypofibrinogenemia, thrombocytopenia, and lactic acidosis [3, 6, 9].

While these three diagnoses are described as separate entities, it is appropriate to consider them as a spectrum of severity of sickle cell hepatopathy. Further, patient presentations suspected of these diagnoses commonly have comorbidities that may muddle the underlying pathology.

With this patient’s clinical features of severe abdominal pain, acute hepatomegaly, anemia without jaundice, renal insufficiency, or bleeding, it is most likely she suffered from acute hepatic sequestration. Furthermore, it can be classified as a severe form of acute hepatic sequestration by the greater than expected total bilirubin levels. While the patient’s alcohol use may have likely contributed to her acute presentation, it is unlikely to be the primary source as she had no evidence of alcoholic liver disease or acute liver failure related to alcohol in her past.

Underlying liver disease represents a significant cause of morbidity and mortality in patients with SCD [9, 10]. Prediction of sickle cell hepatopathy may represent a potential opportunity for risk reduction. Associated risk factors for sickle cell hepatopathy include male sex, sickle cell A (SCA) genotype, lower fetal hemoglobin (HbF), frequent transfusions, elevated gamma-glutamyl transferase (GGT) values, and abnormal liver ultrasound and stiffness [11]. Zinc deficiency has been recognized as a predictive factor for sickle cell hepatopathy which has been hypothesized to be related to its antioxidant and anti-inflammatory properties [12]. However, further studies are required to investigate zinc deficiency correction and its effect on sickle cell hepatopathy. Primary treatment for hepatic sequestration is red cell and exchange transfusions along with management of contributing comorbidities, sickle cell crises, and symptomatic management of encephalopathy [5, 13-16]. Adults commonly have severe degrees of sickle cell hepatopathy with higher rates of mortality compared to adolescents [5]. Effective treatment with exchange transfusions in initial episodes has been associated with improved mortality [5]. While multiple comorbidities and coagulopathy prevented liver biopsy, acute sickle cell hepatopathy may be a contraindication in itself [17]. In end-stage liver disease, transplantation may be considered in the context of the patient’s clinical status although this intervention is associated with high mortality and is not yet proven to be effective [18-20].

​There is a paucity of reported cases and literature regarding sickle cell hepatopathy and acute hepatic sequestration which is likely a consequence of the underrecognition of this constellation of diseases as well as comorbidities that conflate the clinical picture. It is crucial to further develop our understanding and management of these conditions [3, 4].

## Conclusions

Sickle cell hepatopathy, an umbrella term for a constellation of sickle cell complications related to the hepatobiliary system, is a rarely discussed condition which may in part be attributed to its underrecognition and the number of confounding comorbidities that conflate its diagnosis. Sickle cell hepatopathy can be described as hyperbilirubinemia and hepatic dysfunction in the setting of acute crisis. The wide spectrum of diseases generalized as sickle cell hepatopathy include acute sickle cell hepatic crisis, acute hepatic sequestration, and acute intrahepatic cholestasis and may be differentiated by the severity of symptoms and laboratory derangements ultimately guiding effective treatment.
